# Forward flight of birds revisited. Part 2: short-term dynamic stability and trim

**DOI:** 10.1098/rsos.140249

**Published:** 2014-10-15

**Authors:** G. Iosilevskii

**Affiliations:** Faculty of Aerospace Engineering, Technion, Haifa 32000, Israel

**Keywords:** dynamic stability, flapping flight, Hill's equation, parametric resonance

## Abstract

Thrust generation by flapping is accompanied by alternating pitching moment. On the down-stroke, it pitches the bird down when the wings are above its centre of gravity and up when they are below; on the up-stroke, the directions reverse. Because the thrust depends not only on the flapping characteristics but also on the angle of attack of the bird's body, interaction between the flapping and body motions may incite a resonance that is similar to the one that causes the swinging of a swing. In fact, it is shown that the equation governing the motion of the bird's body in flapping flight resembles the equation governing the motion of a pendulum with periodically changing length. Large flapping amplitude, low flapping frequency, and excessive tilt of the flapping plane may incite the resonance; coordinated fore–aft motion, that uses the lift to cancel out the moment generated by the thrust, suppresses it. It is probably incited by the tumbler pigeon in its remarkable display of aerobatics. The fore–aft motion that cancels the pitching moment makes the wing tip draw a figure of eight relative to the bird's body when the wings are un-swept, and a ring when the wings are swept back and fold during the upstroke.

## 2. Introduction

At a first glance, the dynamic stability in flapping flight should not be any different from the dynamic stability in non-flapping flight. After all, the periodic lift, thrust and pitching moment generated by the wings can be viewed as periodic perturbations to the nominal (non-flapping) state. If the latter is stable, the bird is stable, flapping or not. Of course, there can be an interaction between flapping and rigid-body natural modes, but if the flapping frequency is large as compared with the rigid-body natural frequencies, the bird should hardly be affected by flapping at all. Essentially, this was the conclusion of Taylor & Thomas [1].

At a second glance, however, things are much more complicated. Thrust generation by flapping is indeed accompanied by periodic pitching moment: on the down-stroke, it pitches the bird down when the wings are above its centre of gravity, and up, when they are below; on the up-stroke, drag replaces thrust, and hence these directions reverse. The problem is that both the thrust and the drag depend on the angle of attack, and hence an interaction between the motion of the bird's body and flapping may incite a (parametric) resonance, similar to the one reported by Taylor & Zbikowski [2] for desert locust (*Schistocerca gregaria*). Analysis of this resonance is the first objective of this study.

If flapping can incite a resonance, the bird needs an active control that operates on the time scale of the flapping period. In principle, active control can be furnished by moving the tail up and down, or by twisting, cambering and sweeping the wings. In forward flight, the tail is closed, and hence cannot be used for control. Periodic wing twist, proportional to the flapping rate, was shown in Part 1 [3] to be the key element in the effective production of thrust. It is therefore unlikely that the periodic twist is also used to control the pitch. We did not find any account on appreciable variations of camber on birds' wings during the flapping cycle—bats are not addressed in this study. It leaves the fore–aft sweeping motion of the wing to fill the function of the primary active control.

Sweeping motion is manifested in the intricate trajectories drawn by a wing tip during the flapping cycle [4]. These trajectories change among species and change with flight conditions. In some cases, they look like an oblique figure of eight; in other cases, they look like a deformed ring, with up-stroke trajectory passing aft of the down-stroke one. *A posteriori*, our obtaining similar trajectories by simply enslaving the sweeping motion to keep the pitch attitude or the angle of attack makes the conclusion that the sweeping motion is used as the primary control in flight plausible. Trim analysis in the flapping flight is the second objective of this study.

Generic equations of motion can be found in any textbook on flight mechanics [5]. In order to formulate them in explicit form, one needs a model relating the aerodynamic forces generated by the wings with parameters characterizing the flapping and body motions. For this study, we have used the model developed in Part 1 [3]. It has the advantage of furnishing the aerodynamic forces in closed analytical form, and it was shown in Part 1 to be sufficiently accurate to capture the correct behaviour of these forces during the flapping cycle. Since this model is central for this study, we implicitly adopt all the notations and the assumptions of Part 1. For completeness of this presentation, they are briefly recapitulated in the next section.

The rest of the manuscript is organized as follows: equations of motion are derived in §4; the aerodynamic derivatives underlying them are explicated in appendix A. The problem of the short-term dynamic stability is addressed in §5; mathematical details underlying the analysis are found in appendix B. Sweeping motion of the wings is studied in §§6–8. Section 9 concludes the paper. Data of all numerical examples shown in this study are concentrated in appendix C. Morphological data of birds that were used to estimate the range of coefficients in the equations of motion is concentrated in appendix D.

## 3. Preliminaries

As already mentioned, we adopt the notation, the assumptions and the aerodynamic model of Part 1 [3] practically ‘as is’. A bird to be considered here has a pair of identical wings; the length of a single wing (semi-span) is *s*; its area is *S*; the aspect ratio (of the two wings together) is *A*=2*s*^2^/*S*. The average flight velocity is *v*; the density of the air in which the bird flies is *ρ*; the acceleration of gravity is *g*. *s*, *v*, *s*/*v*, *v*/*s*, *ρSs*, *ρSs*^3^, *ρsv*^2^, *ρSv*^2^ and *ρSv*^2^*s* will serve as convenient units of length, velocity, time, frequency, mass, moment of inertia, force per unit span, force and moment, respectively. Note that although *S* is half the quantity commonly used as the wing area, the unit of force is standard. Use of dimensionless quantities will be implicitly understood hereafter. Should a dimensional quantity be required (other than *ρ*, *v*, *g*, *s* and *S*), it will be marked by an asterisk. A list of nomenclature can be found in table 1.

**Table 1.** Nomenclature.

fundamental quantities	
*g*	acceleration of gravity
*S*	area of one wing (left or right)
*s*	semi-span
*v*	flight velocity
*ρ*	air density

fundamental units	
*s*	length
*v*	velocity
*s*/*v*	time
*v*/*s*	frequency
*ρSs*	mass
*ρSs*^3^	moment of inertia
*ρsv*^2^	force per unit span
*ρSv*^2^	force
*ρSv*^2^*s*	moment

non-dimensional quantities	
*A*	aspect ratio
*A*_1_, *A*_2_, …	functions of the aspect ratio; they are defined in (A 4)
*a*	lift slope coefficient of the wing's section (2*π*)
*b*_*n*_	Fourier coefficients (n=−∞,… ,∞); they are introduced in (5.8)
*c*, *c*_0_	local chord, root cord
*D*^*w*^,$D_{0}^{w}$,*D*^*w*^_*i*_	drag of the wing—total, parasite and induced
*f*	flapping frequency
*g*_*s*_	linear damping coefficient in (4.13) and (4.16); it is defined in (4.18)
*H*	determinant
*h*	vertical displacement of the wing's root from a straight path
*I*_11_, *I*_13_	constants, –1/3 and –1/5, respectively
*I*_*y*_	moment of inertia about the centre of gravity
*K*_1_, *K*_4_, *K*_5_	functions of the aspect ratio, see (A 12)
*k*_1_, *k*_4_, *k*_5_	coefficients in (A 12)
*k*_*ψ*1_, *k*_*ψ*2_	constants in (6.1)
*L*	lift
*M*	pitching moment
*m*	mass
*R*_*y*_	reduced radius of gyration, *R*_*y*_=2*r*_*y*_/*c*_0_; it is introduced in (5.19)
*r*_*y*_	radius of gyration; see (3.1)
*t*	time
$\tilde{t}$	reduced time, $\tilde{t}=\omega t$; it is introduced in (5.7)
*x*_*cg*_	longitudinal centre-of-gravity position measured from the quarter-chord of the wing's root, positive aft
*y*_*cp*_	span-wise position of the centre of pressure; it is defined in (7.4)
*α*	angle-of-attack of the bird's body; it is defined in (4.11)
*α*_*g*_, *α*_*g*0_	twist angle and its value at the root (shoulder)
*γ*_*s*_	damping ratio; *γ*_*s*_=*g*_*s*_/*ω*
*δ*_1_, *δ*_2_	constants in (5.7); they are defined in (5.5) and (5.6)
*δ*_*mn*_	Kronecker's delta
*ε*	twist parameter; it is introduced in (3.2)
*μ*	Floquet's exponent; it is introduced in (5.8)
*η*	reduced heave velocity; it is defined in (4.10)
*κ*_*s*_	frequency ratio; $\kappa_{s}={\bar{\omega }}_{s}/\omega$
${\tilde{\kappa }}_{s}$	combination of *κ*_*s*_ and *γ*_*s*_; it is introduced in (B 3)
λ	sweep angle
*τ*	body angle relative to the average flight path
*Φ*_1_, *Φ*_2_	combinations of parameters; they are defined in (5.21) and (5.22)
*ϕ*, *ϕ*_0_	flapping angle and flapping amplitude
*Ψ*_1_,*Ψ*_2_	instability boundaries; they are introduced in (5.16)
*ψ*_1_,*ψ*_2_	constants in (5.7); they are defined in (5.3) and (5.3)
*ω*	flapping angular frequency
${\bar{\omega }}_{s}$	natural frequency of the short-period mode; it is defined in (4.17)

special symbols	
…*	non-fundamental dimensional quantity
${\text{… }}^{\prime },\;{\text{… }}^{\prime \prime },\;\tilde{\cdot \;\cdot \;\cdot }$	modified quantity
…^bt^,…^w^,…^d^	body and tail, wing, parasite drag
$\begin{array}{c} \text{˙}\\ \text{…} \end{array}$	derivative with respect to the (reduced) time
$\begin{array}{c} \text{---}\\ \text{…} \end{array}$	depends on characteristics of the adjoint flight
…_,…_	partial derivative with respect to parameters following the comma (Kronecker's delta)
	is an exception)
…_*s*_	short period

The reduced mass of the bird is *m* (this is the single significant deviation from the notation of Part 1, where *m* was dimensional, rather than reduced, mass). For the sake of simplicity, the mass of the wings is neglected. The centre of gravity is located at the distance *x*_*cg*_ aft of the quarter-chord at the root, and at the same height. The reduced moment of inertia about the centre of gravity is

3.1 $$I_{y}=mr_{y}^{2}\;,$$

where *r*_*y*_ is the respective (reduced) radius of gyration.

The bird's body is allowed to pitch and heave; relative to the body, the wings are allowed to flap, sweep fore and aft, and twist. It is assumed that the wing twists in such a way that its sections do not deform and remain parallel to each other; moreover, the twist axis crosses all sections at their respective quarter-chord points and remains straight at all times. The sweep angle of the twist axis is λ (positive aft), flapping angle is *ϕ* (positive down); the twist angle is *α*_*g*_ (positive for leading edge up); pitch angle is *τ* (positive for nose up); vertical translation of the bird's body (and hence of the twist axis) is *h*. Following equations (3.1) and (5.10) of Part 1, it is assumed that the twist varies linearly along the span; moreover, it follows the flapping rate $\dot{\phi }$ with

3.2 $$\alpha_{g}=\alpha_{g0}-\dot{\phi }\varepsilon \left|y\right|,$$

where the over-dot marks a derivative with respect to time, *y*∈(−1,1) is the span-wise coordinate, *ε*∈(0,1) is a certain proportionality coefficient, and *α*_*g*0_ is the wing twist at the shoulder that remains constant throughout the flapping cycle. The flapping-rate-proportional twist, embodied in the second term on the right, was shown in Part 1 to play a central role in making the flapping propulsion aerodynamically efficient.

In order to comply with the restrictions of the aerodynamic theory developed in Part 1, it is assumed that the wing has an elliptical plan-form, with the chord length prescribed by

3.3 $$c=\frac{8}{\pi A}\sqrt{1-y^{2}}.$$

It is also assumed that *A*^−1^, *α*_*g*0_, *τ*, $\dot{h}$, *ϕ*, $\dot{\phi }$, λ and $\dot{\lambda }$ are small when compared with unity. It was shown in Part 1 that under this assumption, *α*_*g*0_ and *τ* are equivalent. Accordingly, without a loss of generality, it can be agreed that when not flapping, the bird is trimmed with *τ*=0, and the necessary angle of attack is furnished by *α*_*g*0_.

## 4. Equations of motion

Intuitively, the longitudinal dynamics of a bird in flapping flight should be characterized by three distinctive time scales, representing the short-period mode, the phugoid mode, and the flapping itself. Short-period mode is manifested in pitch and heave oscillations with practically no changes in airspeed; phugoid mode is manifested in pitch, heave and airspeed oscillations together ([1] and [5, pp. 167–169]). The longest of the three is the time scale of the phugoid mode, a few seconds (if this mode is stable, its period is estimated as $\pi \sqrt{2}v/g)$. The shortest of the three is probably the flapping period, a few tenths of a second (appendix D). With an order of magnitude separating the shortest and the longest time scales, one may expect that the interaction between the two is small, and hence the flight velocity can be practically assumed constant over the flapping period [1].

With constant flight velocity and nominally horizontal flight, the longitudinal dynamics is governed by the pair of equations

4.1 $$I_{y}\ddot{\tau }=M$$

and

4.2 $$m\ddot{h}=L-\bar{L},$$

where *M* is the pitching moment about the centre of gravity, *L* is the lift and the over-bar denotes the respective quantity in the *adjoint* non-flapping flight (§5.1 in Part 1). In the context of this paper, it is formally defined as the flight at the same velocity and with the same *α*_g0_, but with

4.3 $$\tau =\phi =\dot{\phi }=\dot{h}=0.$$

This definition is consistent with the definition (5.5) of Part 1 as long as the flapping amplitude is sufficiently small. It is implicitly assumed that in the adjoint flight, $\bar{L}$ exactly offsets weight:

4.4 $$\bar{L}=\frac{msg}{v^{2}},$$

and that the bird trims out with sweep angle $\bar{\lambda }$:

4.5 $$\bar{M}=0\;\mathrm{at}\;\lambda =\bar{\lambda }.$$

Based on the aerodynamic model of Part 1, it is shown in appendix A that *L* and *M* can be written in the following symbolic forms:

4.6 $$\begin{array}{rl} L & =\bar{L}+L_{,\alpha }(\tau -\dot{h})+L_{,\dot{\phi }}\dot{\phi }, \end{array}$$

4.7 $$\begin{array}{rl} M & =\left(M_{,\lambda \alpha }(\alpha_{g0}+\tau -\dot{h})+M_{,\lambda \dot{\phi }}\dot{\phi }\right)(\lambda -\bar{\lambda }+\dot{h}\phi )+M_{,\phi \alpha^{2}}\phi (\tau -\dot{h})(2\alpha_{g0}+\tau -\dot{h})\\ & \;+{\bar{M}}_{,\alpha }(\tau -\dot{h})+M_{,\dot{\tau }}\dot{\tau }+M_{,D\phi }{\bar{D}}^{w}\phi +{\bar{M}}_{,\dot{\phi }}\dot{\phi }+M_{,\alpha \phi \dot{\phi }}(\alpha_{g0}+\tau -\dot{h})\phi \dot{\phi }+M_{,\phi {\dot{\phi }}^{2}}\phi {\dot{\phi }}^{2}, \end{array}$$

where *L*_,*α*_, $L_{,\dot{\phi }}$, *M*_,*λα*_, $M_{,\lambda \dot{\phi }}$, *M*_,*ϕα*^2^_, $M_{,\alpha \phi \dot{\phi }}$, $M_{,\phi {\dot{\phi }}^{2}}$, *M*_,*Dϕ*_, ${\bar{M}}_{,\alpha }$, $M_{,\dot{\tau }}$ and ${\bar{M}}_{,\dot{\phi }}$ are the respective partial derivatives (with respect to the variable(s) following the comma), and ${\bar{D}}^{w}$ is the drag of the wing in the adjoint flight; the derivatives that depend on $\bar{\lambda }$ have been marked by the over-bars. Explicit expressions for these derivatives, which are based on the model of Part 1, can be found in appendix A. In principle, however, most (if not all) of these derivative could have been extracted from experimental measurements, as in Taylor & Zbikowski [2] or from numerical simulations, as in Wu & Sun [6].

Substituting (4.7) in (4.1) and (4.6) in (4.2) yields the equations of motion in explicit form:

4.8 $$\begin{array}{rl} & I_{y}\ddot{\tau }-M_{,\dot{\tau }}\dot{\tau }-\left({\bar{M}}_{,\alpha }+M_{,\lambda \alpha }(\lambda -\bar{\lambda }+\dot{h}\phi )+M_{,\phi \alpha^{2}}\phi (2\alpha_{g0}+\tau -\dot{h})+M_{,\alpha \phi \dot{\phi }}\phi \dot{\phi }\right)(\tau -\dot{h})\\ & \;\;=(M_{,\lambda \alpha }\alpha_{g0}+M_{,\lambda \dot{\phi }}\dot{\phi })(\lambda -\bar{\lambda }+\dot{h}\phi )+M_{,D\phi }{\bar{D}}^{w}\phi +{\bar{M}}_{,\dot{\phi }}\dot{\phi }+M_{,\alpha \phi \dot{\phi }}\alpha_{g0}\phi \dot{\phi }+M_{,\phi {\dot{\phi }}^{2}}\phi {\dot{\phi }}^{2}, \end{array}$$

4.9 $$\begin{array}{rl} & m\ddot{h}=L_{,\alpha }(\tau -\dot{h})+L_{,\dot{\phi }}\dot{\phi }. \end{array}$$

In this particular form, they will be used in §8; two additional forms will be needed for §§5–7. To derive the first, we will need the reduced heave velocity,

4.10 $$\eta =\frac{m\dot{h}}{L_{,\alpha }};$$

to derive the second, we will need the angle of attack of the bird's body,

4.11 $$\alpha =\tau -\dot{h}=\tau -\frac{\eta L_{,\alpha }}{m}.$$

The first form comprises the variant

4.12 $$\tau =\dot{\eta }+\frac{L_{,\alpha }}{m}\eta -\frac{L_{,\dot{\phi }}}{L_{,\alpha }}\dot{\phi }$$

of (4.9), and its combination with (4.8),

4.13 $$\begin{array}{rl} & I_{y}\overset{\ldots }{\eta }+I_{y}\left(\frac{L_{,\alpha }}{m}-\frac{M_{,\dot{\tau }}}{I_{y}}\right)\ddot{\eta }-\left({\bar{M}}_{,\alpha }+M_{,\dot{\tau }}\frac{L_{,\alpha }}{m}+M_{,\lambda \alpha }\left(\lambda -\bar{\lambda }\right)+M_{,\phi \alpha^{2}}2\alpha_{g0}\phi +M_{,\alpha \phi \dot{\phi }}^{\prime \prime }\phi \dot{\phi }\right)\dot{\eta }\\ & \;-(M_{,\lambda \alpha }\alpha_{g0}+M_{,\lambda \dot{\phi }}^{\prime \prime }\dot{\phi })\frac{L_{,\alpha }}{m}\phi \eta -\left(M_{,\phi \alpha^{2}}{\dot{\eta }}^{2}+M_{,\lambda \alpha }\frac{L_{,\alpha }}{m}\eta \dot{\eta }\right)\phi \\ & =(M_{,\lambda \alpha }\alpha_{g0}+M_{,\lambda \dot{\phi }}^{\prime \prime }\dot{\phi })\left(\lambda -\bar{\lambda }\right)+I_{y}\frac{L_{,\dot{\phi }}}{L_{,\alpha }}\overset{\ldots }{\phi }-M_{,\dot{\tau }}\frac{L_{,\dot{\phi }}}{L_{,\alpha }}\ddot{\phi }+M_{,D\phi }{\bar{D}}^{w}\phi +{\bar{M}}_{,\dot{\phi }}^{\prime \prime }\dot{\phi }+M_{,\alpha \phi \dot{\phi }}^{\prime \prime }\alpha_{g0}\phi \dot{\phi }+M_{,\phi {\dot{\phi }}^{2}}^{\prime \prime }\phi {\dot{\phi }}^{2}; \end{array}$$

the derivatives $M_{,\alpha \phi \dot{\phi }}^{\prime \prime }$, $M_{,\phi {\dot{\phi }}^{2}}^{\prime \prime }$, $M_{,\lambda \dot{\phi }}^{\prime \prime }$ and ${\bar{M^{\prime \prime }}}_{,\dot{\phi }}$ are found in (A 27)–(A 30). Given $\lambda -\bar{\lambda }$ and *ϕ* as functions of time, equation (4.13) can be solved for *η*; in turn, once *η* is known, *τ* follows by (4.12). Equation (4.13) is the basis for the next two sections.

The second form of the equations includes the combination

4.14 $$m\ddot{h}=L_{,\alpha }\alpha +L_{,\dot{\phi }}\dot{\phi }$$

of (4.11) with (4.9), and the combination of its corollary,

4.15 $$\dot{\tau }=\dot{\alpha }+\ddot{h}=\dot{\alpha }+\frac{L_{,\alpha }}{m}\alpha +\frac{L_{,\dot{\phi }}}{m}\dot{\phi }$$

(which follows by (4.11)) with (4.8),

4.16 $$\begin{array}{rl} & I_{y}\ddot{\alpha }+I_{y}\left(\frac{L_{,\alpha }}{m}-\frac{M_{,\dot{\tau }}}{I_{y}}\right)\dot{\alpha }-\left({\bar{M}}_{,\alpha }+M_{,\dot{\tau }}\frac{L_{,\alpha }}{m}+M_{,\lambda \alpha }(\lambda -\bar{\lambda }+\dot{h}\phi )+M_{,\phi \alpha^{2}}2\alpha_{g0}\phi +M_{,\alpha \phi \dot{\phi }}\phi \dot{\phi }\right)\alpha -M_{,\phi \alpha^{2}}\phi \alpha^{2}\\ & \;=-\frac{I_{y}}{m}L_{,\dot{\phi }}\ddot{\phi }+(M_{,\lambda \alpha }\alpha_{g0}+M_{,\lambda \dot{\phi }}\dot{\phi })(\lambda -\bar{\lambda }+\dot{h}\phi )+M_{,D\phi }{\bar{D}}^{w}\phi +{\bar{M}}_{,\dot{\phi }}^{\prime }\dot{\phi }+M_{,\alpha \phi \dot{\phi }}\alpha_{g0}\phi \dot{\phi }+M_{,\phi {\dot{\phi }}^{2}}\phi {\dot{\phi }}^{2}; \end{array}$$

the derivative ${\bar{M}}_{,\dot{\phi }}^{\prime }$ is found in (A 31). This form will be used in §7.

The combinations

4.17 $${\bar{\omega }}_{s}^{2}=-\left(\frac{{\bar{M}}_{,\alpha }}{L_{,\alpha }}+\frac{M_{,\dot{\tau }}}{m}\right)\frac{L_{,\alpha }}{I_{y}}$$

and

4.18 $$2g_{s}=\frac{L_{,\alpha }}{m}-\frac{M_{,\dot{\tau }}}{I_{y}}$$

of partial derivatives appearing in the second and the third terms on the left-hand sides of (4.13) and (4.16) (they appear multiplied by *I*_*y*_) will be identified with the natural frequency and the damping of the short-period mode [5, p. 175] in the adjoint non-flapping flight. We assume that both ${\bar{\omega }}_{s}^{2}$ and *g*_*s*_ are real and positive—if they were not, the short-period mode would have been unstable, and hence incompatible with the assumptions underlying this short-term stability analysis. There are many arguments that can be brought in support of this statement. Suffice it to say that obtaining an unstable short-period mode contradicts the initial assumption made on its characteristic time scale, and variations in airspeed can no longer be ignored.

## 5. Flapping resonance

We would like to demonstrate that without a coordinated fore–aft sweeping motion of the wings, flapping flight could be dynamically unstable. To this end, we set

5.1 $$\lambda =\bar{\lambda }$$

throughout the flapping cycle and assume that the response in heave is sufficiently small to justify the neglect of the two nonlinear terms on the left-hand side of (4.13). The solution of this linearized version of (4.13) is a combination of homogeneous and particular solutions. Since the right-hand side of this equation does not vanish at all times during the flapping period, if we could demonstrate that its homogeneous part admits a diverging solution, the initial proposition would have been established. The details follow.

For the sake of simplicity, we assume that the flapping is harmonic with amplitude *ϕ*_0_ and (reduced) angular frequency *ω*:

5.2 $$\phi =\phi_{0}\sin\omega t.$$

Introducing $\tilde{t}=\omega t$, *γ*_*s*_=*g*_*s*_/*ω*, $\kappa_{s}={\bar{\omega }}_{s}/\omega$,

5.3 $$\begin{array}{rl} \psi_{2}^{2} & =\frac{M_{,\alpha \phi \dot{\phi }}^{\prime \prime }\phi_{0}^{2}}{4I_{y}\omega }, \end{array}$$

5.4 $$\begin{array}{rl} \psi_{1}^{2} & =-\frac{M_{,\phi \alpha^{2}}\phi_{0}\alpha_{g0}}{I_{y}\omega^{2}}, \end{array}$$

5.5 $$\begin{array}{rl} \delta_{2}^{2} & =-\frac{L_{,\alpha }}{m\omega }\frac{M_{,\lambda \dot{\phi }}^{\prime \prime }\phi_{0}^{2}}{4I_{y}\omega } \end{array}$$

and

5.6 $$\begin{array}{rl} \;\delta_{1}^{2} & =-\frac{L_{,\alpha }}{m\omega }\frac{M_{,\lambda \alpha }\phi_{0}\alpha_{g0}}{2I_{y}\omega^{2}}, \end{array}$$

together with (4.17), (4.18), (5.1)–(5.6) in the homogeneous part of (4.13), allows bringing it into the form:

5.7 $$\frac{d^{3}\eta }{d{\tilde{t}}^{3}}+2\gamma_{s}\frac{d^{2}\eta }{d{\tilde{t}}^{2}}+(\kappa_{s}^{2}+2\psi_{1}^{2}\sin\tilde{t}-2\psi_{2}^{2}\sin2\tilde{t})\frac{d\eta }{d\tilde{t}}+(2\delta_{1}^{2}\sin\tilde{t}+2\delta_{2}^{2}\sin2\tilde{t})\eta =0;$$

the nonlinear terms that have been neglected in due course are $\psi_{1}^{2}(\omega /\alpha_{g0}){\left(d\eta /d\tilde{t}\right)}^{2}\sin\tilde{t}$ and $\delta_{1}^{2}(\omega /\alpha_{g0})(d\eta^{2}/d\tilde{t})\sin\tilde{t}$.

Equation (5.7) resembles Hill's equation [7, p. 405], whereupon we guess its solution in the same form as Floquet's solution of the latter [7, pp. 412–417]:

5.8 $$\eta =-i\sum_{n=-\infty }^{\infty }\frac{b_{n}e^{i\left(\mu +i\gamma_{s}+n\right)\tilde{t}}}{\mu +i\gamma_{s}+n}\;,$$

where *b*'s depend on the initial conditions and *μ* is Floquet's exponent. *b*'s satisfy the recurrence relation

5.9 $$\begin{array}{r} \left(-i\psi_{2}^{2}+\frac{\delta_{2}^{2}}{\mu +i\gamma_{s}+n+2}\right)b_{n+2}+\left(i\psi_{1}^{2}+\frac{\delta_{1}^{2}}{\mu +i\gamma_{s}+n+1}\right)b_{n+1}+\left(-{\left(\mu +n\right)}^{2}+(\kappa_{s}^{2}-\gamma_{s}^{2})\right)b_{n}\\ \;+\left(-i\psi_{1}^{2}-\frac{\delta_{1}^{2}}{\mu +i\gamma_{s}+n-1}\right)b_{n-1}+\left(i\psi_{2}^{2}-\frac{\delta_{2}^{2}}{\mu +i\gamma_{s}+n-2}\right)b_{n-2}=0; \end{array}$$

*μ* makes the determinant of the infinite, 5-diagonal matrix, formed on the coefficients of *b*'s in (5.9), vanish:

5.10 $$H(\mu ,\sqrt{\kappa_{s}^{2}-\gamma_{s}^{2}},\gamma_{s},\psi_{2},\psi_{1},\delta_{2},\delta_{1})=0.$$

Equation (5.9) is obtained by substituting (5.8) in (5.7); without (5.10), the relations in (5.9) would have admitted only a trivial solution.

**Figure 1.**
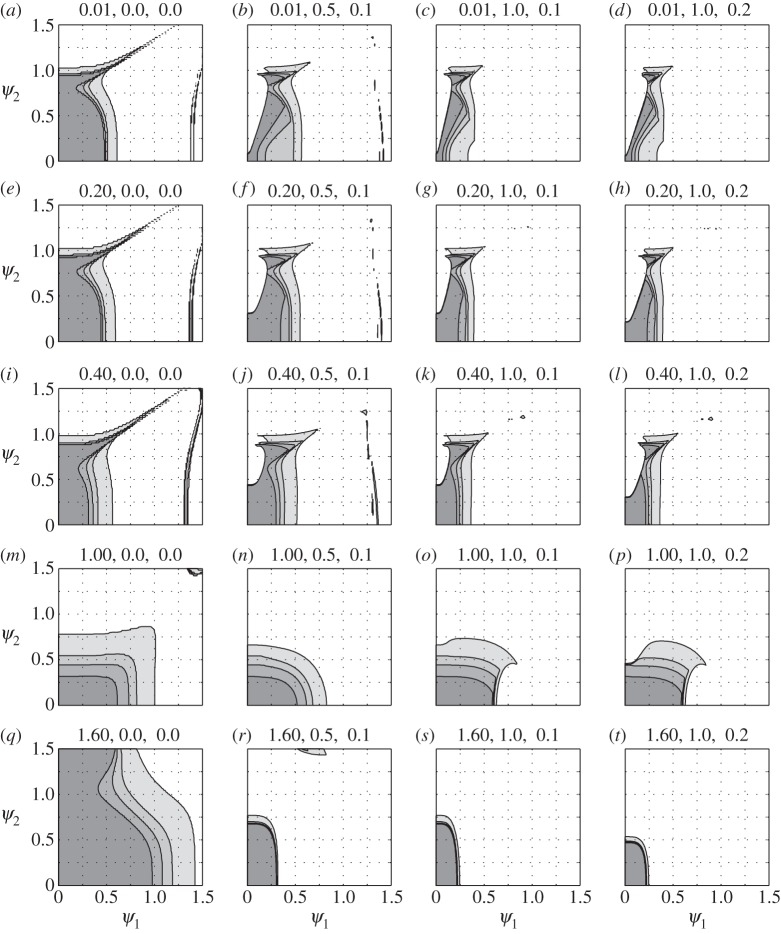
Shaded areas mark combinations of *ψ*_1_ and *ψ*_2_ where solutions of (5.7) are bounded with different values of *γ*_*s*_, *κ*_*s*_, *δ*_1_ and *δ*_2_. The numbers in the title of each sub-figure are the values of *κ*_*s*_, *δ*_1_/*ψ*_1_ and *δ*_2_/*ψ*_2_, respectively; four values of *γ*_*s*_, 0.05, 0.1, 0.15 and 0.3, are colour-coded, between dark and light shades of grey. For example, with *γ*_*s*_=0.3, the stable region in figure 1*m* is roughly the rectangle (0,1)×(0,0.8); with *γ*_*s*_=0.05, it shrinks to (0,0.6)×(0,0.3).

By mapping the solutions of (5.10) over possible combinations of *γ*_*s*_, *κ*_*s*_, *ψ*_1_, *ψ*_2_, *δ*_1_ and *δ*_2_ one can identify those combinations for which Im *μ*>−*γ*_*s*_; that is, one can identify the conditions for which the solutions of (5.7) are bounded. Details can be found in appendix B; the outcome is shown in figure 1. Judging by this figure, a rational description of those conditions is hardly possible. Nonetheless, a few conclusions can be made based on explicit form of the parameters in (5.7):

5.11 $$\begin{array}{rl} 2\gamma_{s} & =\frac{L_{,\alpha }}{m\omega }-\frac{M_{,\dot{\tau }}}{m\omega r_{y}^{2}}, \end{array}$$

5.12 $$\begin{array}{rl} \psi_{2}^{2} & =\frac{L_{,\alpha }}{m\omega }\frac{\phi_{0}^{2}}{16r_{y}^{2}}\left(1-\left(1-\varepsilon \right)\left(k_{4}-\frac{128}{9\pi^{2}}\right)A_{1}\right), \end{array}$$

5.13 $$\begin{array}{rl} \psi_{1}^{2} & =\frac{L_{,\alpha }}{m\omega }\frac{\phi_{0}^{2}}{16r_{y}^{2}}\frac{\alpha_{g0}}{\omega \phi_{0}}\frac{64A_{1}}{3\pi }, \end{array}$$

5.14 $$\begin{array}{rl} \frac{\delta_{2}^{2}}{\psi_{2}^{2}} & =\frac{L_{,\alpha }}{m\omega }\frac{(64/9\pi^{2})(k_{1}-1)\left(1-\varepsilon \right)}{1-\left(1-\varepsilon \right)\left(k_{4}-(128/9\pi^{2})\right)A_{1}} \end{array}$$

and

5.15 $$\begin{array}{rl} \frac{\delta_{1}^{2}}{\psi_{1}^{2}} & =\frac{L_{,\alpha }}{m\omega }\frac{1}{2A_{1}}, \end{array}$$

and physical data found in appendix D. Equation (5.11) follows (4.18) by (3.1) (note the definition of *γ*_*s*_ in the line preceding (5.3)); (5.12) follows (5.3) by (A 27) , (3.1), (A 9), (A 11) and (A 3); (5.13) follows (5.4) by (3.1) and (A 11); (5.14) follows (5.3) and (5.5) by (A 27), (A 29), (A 8), (A 9), (A 11) and (A 3); (5.15) follows (5.6) and (5.4) by (A 11) and (A 7). *A*_1_ is given by (A 4); *k*_1_≈1.29 and *k*_4_≈2.29 from (A 12).

Here *δ*_2_/*ψ*_2_ and *δ*_1_/*ψ*_1_ are only weakly dependent on flight conditions (through the square root of the flapping frequency). For most species of the birds compiled in appendix D, *δ*_1_/*ψ*_1_∈(0.5,1) at cruise; moreover, assuming *ε*=0.5, *δ*_2_/*ψ*_2_∈(0.1,0.2). Three combinations of *δ*_1_/*ψ*_1_ and *δ*_2_/*ψ*_2_ from these ranges are shown in the right three columns of figure 1.

$M_{,\dot{\tau }}$ is contributed mainly by the tail. Since the tail is closed at cruise, one may expect this derivative to be small. Consequently, the value of *γ*_*s*_ is determined largely by *L*_,*α*_/2*mω*. For most of the species of birds compiled in appendix D, *L*_,*α*_/2*mω*∈(0.05,0.3). Shaded areas in figure 1 reflect values of *γ*_*s*_ in this range.

The value of *κ*_*s*_ depends on the stability margin and the flapping frequency. The stability margin of a bird is probably small—otherwise large pitching up moment would have been required to trim the bird in the adjoint flight [8]. Small stability margin implies small *ω*_*s*_, and hence one may expect that the flapping frequency will be higher than the frequency of the short-period mode in the adjoint flight. Three cases with *κ*_*s*_<1 are shown in the first three rows in figure 1.

Based on the first three rows in the right three columns of figure 1, one can state that with the exception of a few singular cases, a bird is unstable if

5.16 $$\psi_{2}>\Psi_{2}\;\mathrm{or}\;\psi_{1}>\Psi_{1},$$

where *Ψ*_1_ and *Ψ*_2_ are, in general, intricate functions of *κ*_*s*_, *γ*_*s*_, *δ*_1_/*ψ*_1_ and *δ*_2_/*ψ*_2_. Towards the following discussion, they will be replaced by their representative values over these nine figures, say, *Ψ*_1_=1 and $\Psi_{2}=\sqrt{0.1}$. Using (5.12), the first criterion in (5.16) translates into

5.17 $$\frac{L_{,\alpha }}{m\omega }\frac{\phi_{0}^{2}}{16r_{y}^{2}}\left(1-\left(1-\varepsilon \right)\left(k_{4}-\frac{128}{9\pi^{2}}\right)A_{1}\right)>\Psi_{2}^{2};$$

using (5.13), (A 17), (A 2) and (4.4), the second one translates into

5.18 $$\frac{\phi_{0}}{r_{y}^{2}}\frac{g}{f^{*2}s}\frac{A_{1}}{3\pi^{3}}>\Psi_{1}^{2},$$

where f*=*ωv*/2*πs* is the dimensional flapping frequency. Whichever criterion applies, a combination of small radius of gyration, slow flapping and large flapping amplitude may lead to resonance.

The data on the radius of gyration among birds is scarce. Exploiting the interpretation of the radius of gyration as half the length of a dumbbell having the same mass and inertia as the body represented by it, we estimate the radius of gyration of a bird to be comparable with half the chord at the wing's root, *c*_0_=8/*πA*—see (3.3). In other words, when writing

5.19 $$r_{y}=R_{y}\frac{c_{0}}{2}=R_{y}\frac{4}{\pi A},$$

*R*_*y*_ will be a parameter of the order of unity. In fact, based on the data of Hedrick & Biewener [9], *R*_*y*_=0.56 for a cockatoo (*Eolophus roseicapillu*s).

Substituting (5.19) in (5.17) and (5.18), the instability criteria (5.16) can be re-formulated as

5.20 $$\phi_{0}>\min\left(\frac{R_{y}\Psi_{2}}{\Phi_{2}},\;\frac{R_{y}^{2}\Psi_{1}^{2}}{\Phi_{1}^{2}}\right),$$

where

5.21 $$\Phi_{2}=\frac{\pi A}{16}\sqrt{\frac{L_{,\alpha }}{m\omega }\left(1-\left(1-\varepsilon \right)\left(k_{4}-\frac{128}{9\pi^{2}}\right)A_{1}\right)}$$

and

5.22 $$\Phi_{1}=\frac{A}{4}\sqrt{\frac{g}{f^{*2}s}\frac{A_{1}}{3\pi }}.$$

**Figure 2.**
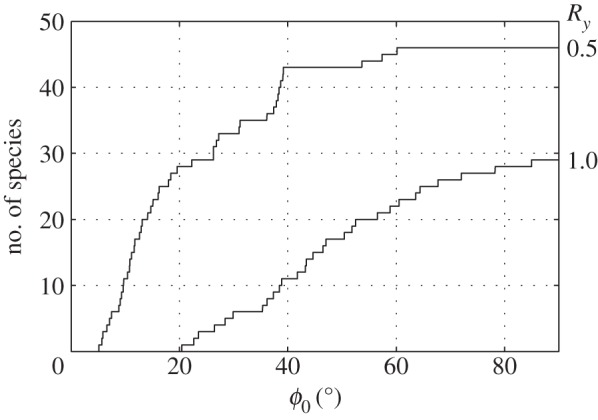
Number of species, from the 46 species that have been compiled in appendix D, that are unstable by criterion (5.20) if the flapping amplitude is *ϕ*_0_. The two lines represent different radii of gyration, *r*_*y*_=4*R*_*y*_/*πA*.

With *Ψ*_2_=1, $\Psi_{1}=\sqrt{0.1}$ and *R*_*y*_=1, it renders 15 out of 46 species of birds compiled in appendix D unstable when the flapping amplitude exceeds 45^°^; with *R*_*y*_=0.5, the number rises to 43 (figure 2). Being based on quite a few assumptions, this conclusion should be treated with due caution. Still, it implies that the resonance is within reach and, regardless of the intrinsic stability of a bird in non-flapping flight, some sort of stabilization during flapping flight is a necessity. For the reasons already mentioned in the Introduction, this stabilization is likely to be furnished by the sweeping motion of the wing.

## 6. Inciting the resonance

We release now the assumption (5.1) that inhibited the wing sweep, and temporarily assume that the sweeping motion is prescribed by

6.1 $$\lambda =\bar{\lambda }+k_{\psi_{2}}\phi \dot{\phi }\frac{M_{,\alpha \phi \dot{\phi }}^{\prime \prime }}{M_{,\lambda \alpha }}+2k_{\psi_{1}}\alpha_{g0}\phi \frac{M_{,\phi \alpha^{2}}}{M_{,\lambda \alpha }},$$

where *k*_*ψ*_2__ and *k*_*ψ*_1__ are certain parameters. This assumption will be released in the next section. Combined with flapping, the fore–aft motion prescribed by (6.1) makes the wing tip draw an oblique figure of eight (the first term makes it a figure of eight; the second term tilts the flapping plane forwards). Replacing (5.1) with (6.1), and repeating the steps leading from (4.13) to (5.7), yields a similar equation, only in which

6.2 $$\psi_{2}^{2}=(1+k_{\psi_{2}})\frac{M_{,\alpha \phi \dot{\phi }}^{\prime \prime }\phi_{0}^{2}}{4I_{y}\omega }$$

and

6.3 $$\psi_{1}^{2}=-(1+k_{\psi_{1}})\frac{M_{,\phi \alpha^{2}}\phi_{0}\alpha_{g0}}{I_{y}\omega^{2}}.$$

Although it is plausible that some combination of *k*_*ψ*_2__ and *k*_*ψ*_1__ will make the bird stable, it is certain that sufficiently large *k*_*ψ*_2__ or *k*_*ψ*_1__ will make it unstable. This conjecture follows by observing the stability regions in the left column of figure 1 (as *k*_*ψ*_2__ and *k*_*ψ*_1__ increase, *δ*_1_/*ψ*_1_ and *δ*_2_/*ψ*_2_ tend to zero). It is plausible that the sweeping-motion-incited resonance is exploited by the tumbler pigeon (*Columba livia*) in its repetitive somersaulting.

Returning to (5.7) one may note that substitution $\bar{t}=\bar{t^{\prime }}+\pi$ changes sign with the terms involving *ψ*_1_ and *δ*_1_. Consequently, the stability analysis of the preceding section applies for positive and negative values of $\psi_{1}^{2}$ alike (only its absolute value counts), and hence the resonance can be incited with large negative values of *k*_*ψ*_1__ as well as with large positive values. In other words, it can be incited by tilting the flapping plane forwards and backwards alike.

All birds tilt the flapping plane backwards during the transition from forward to hovering flights [4]. In view of the above, it can incite the resonance. Since the tilt of the flapping plane is dictated by the performance requirements—the thrust is needed for lift—it is plausible that during these stages of flight the tail replaces the wing as the primary active control. In fact, the tail has been observed to open up with decreasing flight speed [4].

## 7. Trim at zero angle of attack

In general, there is infinite number of flight strategies that can be realized using active control. Here, we consider the most obvious two: keeping the angle of attack zero throughout the flapping cycle, and keeping the pitch angle zero throughout the cycle. Starting with the first strategy, the sweeping motion that makes

7.1 α=0

is the one that makes the right-hand side of (4.16) vanish:

7.2 $$\lambda -\bar{\lambda }=-\dot{h}\phi -\frac{-(I_{y}/m)L_{,\dot{\phi }}\ddot{\phi }+M_{,\dot{\phi }}^{\prime }\dot{\phi }+M_{,D\phi }{\bar{D}}^{w}\phi +(M_{,\alpha \phi \dot{\phi }}\alpha_{g,0}+M_{,\phi {\dot{\phi }}^{2}}\dot{\phi })\phi \dot{\phi }}{M_{,\lambda \dot{\phi }}\dot{\phi }+M_{,\lambda \alpha }\alpha_{g,0}}.$$

Here,

7.3 $$\dot{h}=\frac{L_{,\dot{\phi }}\phi }{m}$$

by (4.14) and (7.1).

By interpretation, the numerator on the right-hand side of (7.2)—if written as a single fraction—is the pitching moment at *α*=0 and $\lambda =\bar{\lambda }$, unmitigated by the fore–aft motion of the wing. To within a sign, the denominator in (7.2) is twice the flapping moment acting on the right wing at *α*=0, (*M*_*x*_)_*α*=0_; this conjecture follows from (A 7) and (A 8) of appendix A, and (4.26) of Part 1. In turn,

7.4 $${\left(M_{x}\right)}_{\alpha =0}=y_{\mathrm{cp}}{\left(L\right)}_{\alpha =0}$$

can be associated with the action of lift, (*L*)_*α*=0_, at the centre of pressure of the right wing,

7.5 $$y_{\mathrm{cp}}={\left(\frac{M_{x}}{L}\right)}_{\alpha =0}=\frac{4}{3\pi }\frac{\alpha_{g0}+(4/3\pi )k_{1}\left(1-\varepsilon \right)\dot{\phi }}{\alpha_{g0}+(4/3\pi )\left(1-\varepsilon \right)\dot{\phi }}.$$

**Figure 3.**
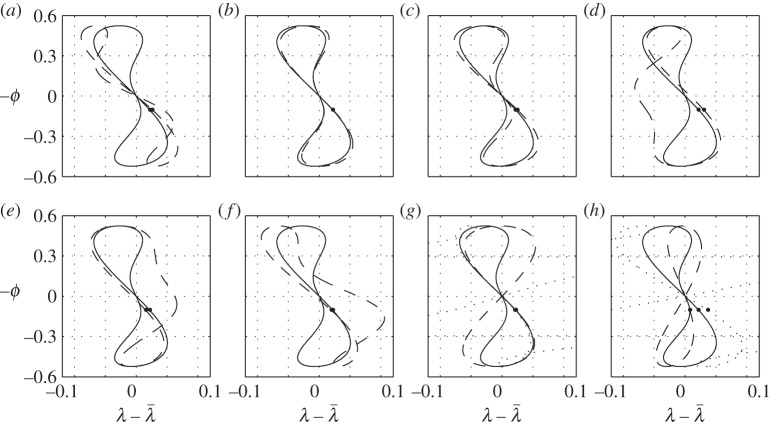
Trajectories of the left wing tip generated with (7.2); flight direction is to the left; the down-stroke starts at the upper left corner and ends at the lower right—it is marked by a small dot. The solid line in all figures represents case 1 of appendix C. Dashed lines show the effects of (*a*) increased inertia, (*b*) increased mass, (*c*) increased aspect ratio, (*d*) aft centre-of-gravity placement with $\bar{\lambda }=0$, (*e*) aft centre-of-gravity placement with $\bar{\lambda }=x_{\mathrm{cg}}3\pi /4$, (*f*) aft centre of gravity and increased inertia combined, (*g*) increased twist and (*h*) decreased frequency. These are cases 2–9 in appendix C, respectively. Dotted lines in (*g*) and (*h*) mark a decreased twist and an increased frequency, respectively (cases 10 and 11).

**Figure 4.**
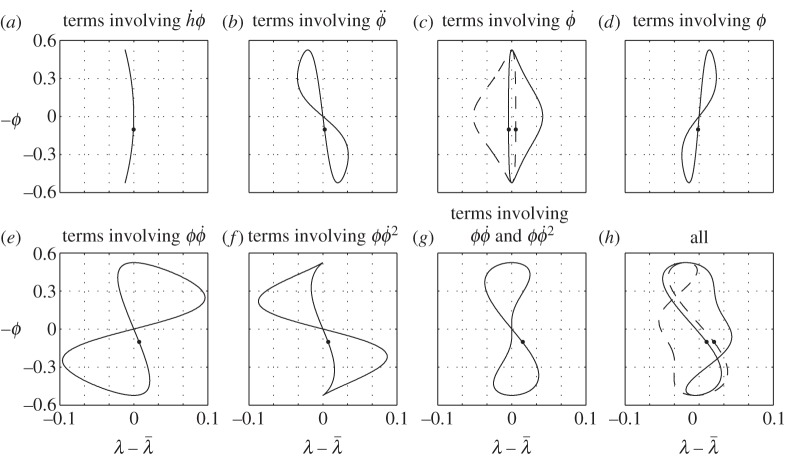
(*a*–*h*) Contributions of the separate terms in (7.2) for cases 5 (dashed line) and 6 (solid line), corresponding to figure 3*d*,*e*. Shown are the trajectories of the left wing tip; flight direction is to the left; down-stroke is marked by a dot.

The expression on the left of (7.5) is just a definition of the centre of pressure (twice the moment generated by a single wing over twice its lift); the expression on the right follows from (4.4), (4.5), (4.24), (4.26) and (4.31) of Part 1. This interpretation of (7.2) elucidates the balancing action: the lift is moved fore-and-aft to counteract the moment created by the thrust and by the drag. A few examples of the wing tip trajectories can be found in figure 3. Individual contributions of the six terms on the right-hand side of (7.2) can be found in figure 4.

The fundamental shape drawn by the wing tip is a deformed figure of eight. The three terms that are responsible for this shape are the first and the last two terms on the right-hand side of (7.2), those involving the combinations $\dot{h}\phi$, $\phi \dot{\phi }$ and $\phi {\dot{\phi }}^{2}$. All three can be associated with the pitching moment created by the thrust. Loosely speaking, thrust is generated by the rotation of the lift vector—it rotates forwards when the wing moves down (either due to flapping or due to heave) and backwards when the wing moves up. Since heave is in phase with the flapping angle (the bird moves up when the wings are down—see (7.3)), the lift rotation due to heave pitches the bird down during the entire flapping cycle. To counteract it, the wing tips draw a crescent shape, moving forward towards the ends of the up-stroke and the down-stroke (figure 4*a*). At the same time, the lift rotation due to flapping creates an alternating moment, pitching the bird down at the beginnings of the up-stroke and the down-stroke, and pitching it up towards their respective ends. To counteract it, the wing tips draw a figure of eight, moving backwards during both the up-stroke and the down-stroke (figure 4*g*).

Modifications to the basic figure of eight come from the remaining three terms. The first one involves $\ddot{\phi }$. This term can be associated with the pitching moment needed to coordinate the pitch with heave in keeping the angle of attack constant. To this end, the pitch angle should be proportional to the heave velocity, and hence to the flapping angle. In turn, it requires the pitching moment to be proportional to the pitch acceleration, and hence to the flapping angle acceleration. It brings the wings forwards (relative to their nominal positions) at the beginning of the down-stroke, and backwards at its end; the down-stroke and the up-stroke are different because the lift is different (figure 4*b*).

The next term involves *ϕ* and ${\bar{D}}^{w}$. It is associated with drag—it pitches the bird up when the wings are up, and down when the wings are down. To counteract the action of this moment, the flapping plane tilts backwards, with the wings backwards when up and forwards when down (figure 4*d*).

The last term involves $\dot{\phi }$. It shapes the figure of eight by moving the down-stroke and the up-stroke legs in the opposite directions (figure 4*c*). When the bird trims out in the adjoint flight with straight wings ($\bar{\lambda }=0$) and aft centre of gravity (*x*_*cg*_>0), the wings shift backwards on the way down and forwards on the way up, effectively blowing up the lower part of the figure of eight and shrinking its upper part (figures 3*d* or 4*h*). With $\bar{\lambda }>0$ and the same centre-of-gravity position, it may be the other way around, and the wings shift forwards on the way down and backwards on the way up (figures 3*e* or 4*h*).

This intricate behaviour is associated with the lift and centre-of-pressure fluctuations during flapping. In the first case ($\bar{\lambda }=0$, *x*_*cg*_>0), the wings' centre of pressure is always forwards of the centre of gravity. The lift increase on the down-stroke creates positive pitching moment about the centre of gravity, and hence the wings should move backwards to compensate; the opposite happens on the upstroke. In the second case, the wings' centre of pressure is not necessarily forwards of the centre of gravity. For example, $\bar{\lambda }=x_{\mathrm{cg}}3\pi /4$ (which implies $M_{0}^{\mathrm{bt}}=0$ by (A 20)) longitudinally aligns the wings' centre of pressure with the centre of gravity (assuming $M_{,\alpha }^{\mathrm{bt}}$ small, it also makes the bird near neutrally stable—see (A 26)). When the bird starts flapping, the wing's centre of pressure moves outwards (and hence backwards) on the down-stroke and inwards (and hence forwards) on the up-stroke—see (7.5). Consequently, the sweep has to be smaller on the way down than on the way up. Moreover, if the wings fold on the way up, the centre of pressure moves further inwards (and hence forwards), magnifying the effect. In fact, it can make the wing tip draw a non-intersecting ring rather than a figure of eight [4].

**Figure 5.**
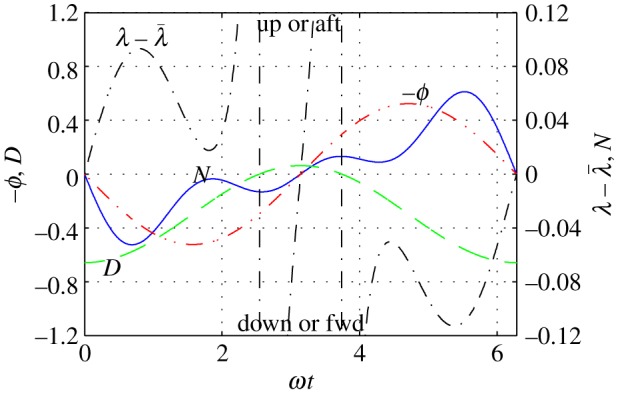
The numerator (*N*, blue solid line) and the denominator (*D*, green dashed line) in (7.2); the flapping angle (*ϕ*, red dash–dot line); and the sweep angle (λ, black dash–dot line). Conditions are those of case 11 in appendix C. During the upstroke (*π*/2<*ωt*<3*π*/2), the unmitigated pitching moment, represented by *N*, is small, but the lift generated by the wing is insufficient to balance it.

When the angle of attack due to flapping is comparable with the average angle of attack, *α*_g0_—either because of high flapping rate or because of small twist or because of small *α*_g0_—the denominator in (7.2) may vanish at certain times during the up-stroke (figures 3*g*,*h* and 5). During these events, it will be impossible to balance the pitching moment with fore–aft adjustment of the wing. A bird has two options here. One is to use the tail for control; in fact, the tail often opens up during vigorous flapping. The other is to do nothing. Since in those cases where the denominator in (7.2) can vanish, the pitching moment is small during the entire upstroke (note the range *π*/2<*ωt*<3*π*/2 in figure 5), a momentary imbalance should be of no consequence.

## 8. Trimming at zero pitch angle

Substituting

8.1 τ=0

in (4.8) yields the sweeping angle required to keep the pitch attitude during flight:

8.2 $$\begin{array}{rl} \lambda -\bar{\lambda } & =-\dot{h}\phi +\frac{M_{,\alpha }\dot{h}-{\bar{M}}_{,\dot{\phi }}\dot{\phi }}{M_{,\lambda \dot{\phi }}\dot{\phi }+M_{,\lambda \alpha }(\alpha_{g0}-\dot{h})}\\ & \;+\frac{-M_{,D\phi }\left(D_{0}^{w}+\pi AA_{1}^{2}{\left(\alpha_{g0}-\dot{h}\right)}^{2}\right)\phi -M_{,\alpha \phi \dot{\phi }}(\alpha_{g0}-\dot{h})\phi \dot{\phi }-M_{,\phi {\dot{\phi }}^{2}}\phi {\dot{\phi }}^{2}}{M_{,\lambda \dot{\phi }}\dot{\phi }+M_{,\lambda \alpha }(\alpha_{g0}-\dot{h})}. \end{array}$$

To obtain (8.2) in this particular form, we have used the identity

8.3 $$\begin{array}{rl} M_{,D\phi }{\bar{D}}^{w}-M_{,\phi \alpha^{2}}\dot{h}(-\dot{h}+2\alpha_{g0}) & =M_{,D\phi }\left(D_{0}^{w}+\pi AA_{1}^{2}\alpha_{g0}^{2}-\pi AA_{1}^{2}\dot{h}(-\dot{h}+2\alpha_{g0})\right)\\ & =M_{,D\phi }\left(D_{0}^{w}+\pi AA_{1}^{2}{\left(\alpha_{g0}-\dot{h}\right)}^{2}\right), \end{array}$$

stemming from (A 14), (A 25), (A 11) and (A 2). In (8.2), $\dot{h}$ satisfies

8.4 $$m\ddot{h}+L_{,\alpha }\dot{h}=L_{,\dot{\phi }}\dot{\phi }$$

by (8.1) and (4.9); hence, if *ϕ*=Re(*ϕ*_0_*e*^*iωt*^),

8.5 h˙=Re(L,ϕ˙ϕ0eiωtimω+L,α).

**Figure 6.**
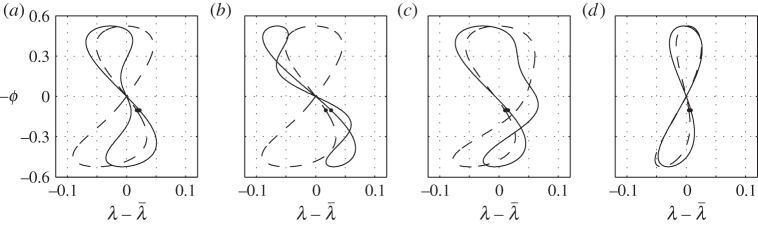
Trajectories of the left wing tip that keep the pitch angle constant (dashed line) when compared with those that keep the angle of attack constant (solid line). Flight direction is to the left; the down-stroke starts at the upper left corner and ends at the lower right—it is marked by a small dot. Shown are (*a*) case 1, (*b*) increased inertia (case 2), (*c*) aft centre-of-gravity placement with $\bar{\lambda }=x_{\mathrm{cg}}3\pi /4$ (case 6) and (*d*) decreased frequency (case 9).

A few examples of wing tip trajectories can be found in figure 6. They look similar to those shown in figure 3, but since there is no need to adjust the pitch angle to heave, less fore–aft motion on the down-stroke is required.

## 9. Concluding remarks

Without active control, an interaction between flapping, pitching and heave can cause a resonance that is similar to the one that causes the swinging of a swing. It may affect all birds that have small inertia in pitch when flapping slowly and with large amplitude, irrespective of their stability margin. This resonance can be suppressed by active control—either with fore–aft sweeping motion of the wing or with up-and-down deflection of the tail. It is most probably incited by a tumbler pigeon in its repetitive somersaulting. It is possibly incited by all birds during the transition from forward to hovering flight, and suppressed by the tail (in this flight regime most birds open their tails). It is suppressed in forward flight (where the tail is closed) by the sweeping motion of the wing. Our obtaining wing tip trajectories, that resemble those observed in birds, by simply enslaving the sweeping motion of the wing to keep either the angle of attack or the pitch attitude supports this conjecture.

Three key elements made this analysis possible. One is the aerodynamic model that was developed in Part 1. It allowed introduction of the aerodynamic derivatives (appendix A), which were instrumental in keeping the length of the associated equations in check. The second element was the theory of the third-order differential equation governing the short-term dynamics of the bird that was developed in appendix B. It allowed obtaining the stability boundaries without running time-consuming numerical solutions of this equation. The third element was in restricting the range of the six parameters of that equation based on physical data (appendix D). It would have been practically impossible to draw any definitive conclusions with six unrestricted parameters.

## Figures and Tables

**Figure 7.**
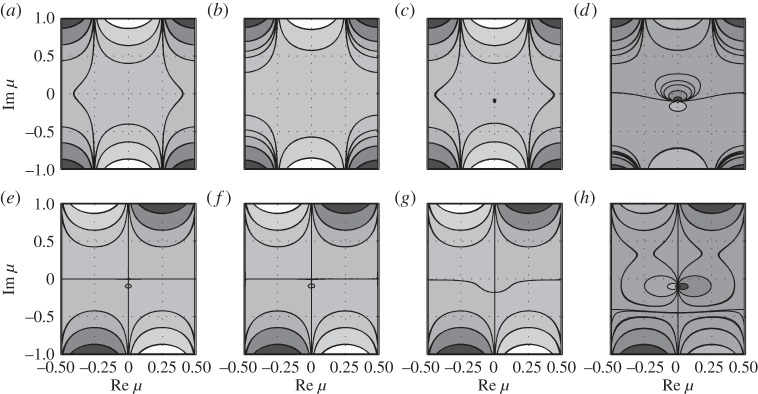
(*a*–*h*) Maps of *H*(*μ*,0.387,0.1,*ψ*_1_,*ψ*_2_,*δ*_1_,*δ*_2_), real part at the top row, imaginary part at the bottom, for four combinations of parameters *ψ*_1_, *ψ*_2_, *δ*_1_ and *δ*_2_. From left to right, the combinations are (0.2, 0.2, 0, 0), (0.8, 0.4, 0, 0), (0.2, 0.2, 1, 0.1) and (0.8, 0.4, 1, 0.1). Contour levels are −4^4^, −4^3^, −4^2^, −4^1^, 0, 4^1^, 4^2^, 4^3^ and 4^4^, colour-coded between the darkest and the lightest shades of grey. Equation (B 11), shown by dashed lines, is graphically indistinguishable. The determinant was computed on an 81×81 matrix (its convergence was verified by halving and doubling the size of the matrix); the coefficients in (B 11) were computed based on five points: *μ*=0 and *μ*=±0.29±0.29*i*.

**Figure 8.**
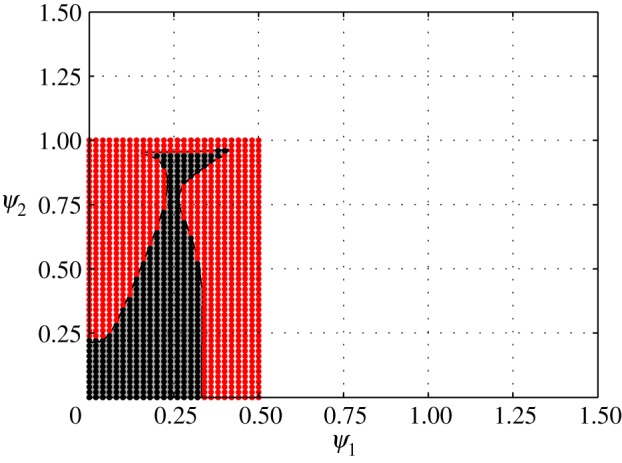
Direct numerical solution of equation (B 1) with *κ*_*s*_=0.2, *γ*_*s*_=0.15, *δ*_1_/*ψ*_1_=1 and *δ*_2_/*ψ*_2_=0.2. Solid line marks the stability boundary found by examining the solutions of (B 22); red and black dots mark diverging and converging solutions of (B 1).

## Appendix A. Lift and pitching moment

In this appendix, we derive explicit expressions for the lift and the pitching moment acting on a bird in flapping flight. The main contributors to both parameters are the wing(s), the body and the tail.

The combined body and tail contribution to the lift is expected to be small. In fact, exploiting the slender body theory, it should be proportional to the ratio of the tail span squared and the wing(s) area. Neglecting this contribution *a priori*, *L* is furnished by the conjunction of (4.4), (4.24) and (5.11) of Part 1 (the last equation applies because of (3.2)). We write it here in the symbolic form:

A 1 $$L=L_{,\alpha }(\alpha_{g0}+\tau -\dot{h})+L_{,\dot{\phi }}\dot{\phi },$$

where

A 2 $$\begin{array}{c} L_{,\alpha }=\pi AA_{1}, \end{array}$$

A 3 $$\begin{array}{c} L_{,\dot{\phi }}=\frac{4}{3}AA_{1}\left(1-\varepsilon \right)=\frac{4}{3\pi }L_{,\alpha }\left(1-\varepsilon \right), \end{array}$$

and in which, with any integer *n*,

A 4 $$A_{n}=\frac{2}{A+2n}.$$

In (A 1), as well as in all subsequent occurrences, a comma in the subscript stands for a partial derivative with respect to the variable(s) following it.

We divide the pitching moment about the centre of gravity, into four constituents:

A 5 $$M=M^{w}+Lx_{\mathrm{cg}}+M^{d}+M^{\mathrm{bt}}.$$

The last one comes from the body and the tail; the first three come from the wing. Among these three, *M*^*w*^ is the pitching moment about the quarter-chord point of the wing's root, contributed by the pressure loads acting on the wing—it is furnished by (4.27) in Part 1 (in which it was denoted *M*_*y*_); *Lx*_*cg*_ shifts this moment to the centre of gravity; *M*^*d*^ is the pitching moment contributed by the parasite drag.

Based on (4.27) of Part 1, and similar to (A 1), the expression for *M*^*w*^ is recast as

A 6 $$\begin{array}{rl} M^{w} & =\left(M_{,\lambda \alpha }(\alpha_{g0}+\tau -\dot{h})+M_{,\lambda \dot{\phi }}\dot{\phi }\right)(\lambda +\dot{h}\phi )\\ & \;+M_{,\alpha \phi \dot{\phi }}(\alpha_{g0}+\tau -\dot{h})\phi \dot{\phi }+M_{,\phi {\dot{\phi }}^{2}}\phi {\dot{\phi }}^{2}+M_{,\phi \alpha^{2}}\phi {\left(\alpha_{g0}+\tau -\dot{h}\right)}^{2}, \end{array}$$

where the respective coefficients (partial derivatives),

A 7 $$\begin{array}{rl} M_{,\lambda \alpha } & =4AA_{1}I_{11}=-\frac{4}{3\pi }L_{,\alpha }, \end{array}$$

A 8 $$\begin{array}{rl} M_{,\lambda \dot{\phi }} & =-\frac{16}{\pi }AK_{1}\left(1-\varepsilon \right)=-\frac{16}{9\pi^{2}}L_{,\alpha }k_{1}\left(1-\varepsilon \right), \end{array}$$

A 9 $$\begin{array}{rl} M_{,\alpha \phi \dot{\phi }} & =\frac{\pi }{4}AA_{1}\left(1-\frac{32}{\pi^{2}}\left(1-\varepsilon \right)K_{4}\right)=\frac{L_{,\alpha }}{4}(1-\left(1-\varepsilon \right)k_{4}A_{1}), \end{array}$$

A 10 $$\begin{array}{rl} M_{,\phi {\dot{\phi }}^{2}} & =-\left(1-\varepsilon \right)AA_{1}I_{11}\left(1+\frac{A_{3}I_{13}}{A_{1}I_{11}}-\frac{32}{\pi^{2}}\frac{\left(1-\varepsilon \right)K_{5}}{A_{1}I_{11}}\right)\\ & =\frac{L_{,\alpha }\left(1-\varepsilon \right)}{3\pi }\left(1+\frac{3A_{3}}{5A_{1}}-\left(1-\varepsilon \right)A_{1}\frac{k_{5}}{3}\right) \end{array}$$

and

A 11 $$\begin{array}{rl} M_{,\phi \alpha^{2}} & =4AA_{1}^{2}I_{11}=-\frac{4}{3\pi }L_{,\alpha }A_{1}, \end{array}$$

are identified by matching between (A 6) and the original expression in Part 1. *I*_11_ and *I*_13_ are actually standard integrals, the former equals −1/3 and the latter equals −1/5. *K*_1_, *K*_4_ and *K*_5_ are functions of the aspect ratio found in (4.28) and (4.29) in Part 1. They are approximated with

A 12 $$K_{1}=k_{1}A_{1}I_{11}^{2},\;K_{4}=\frac{\pi^{2}}{32}k_{4}A_{1},\;K_{5}=-\frac{\pi^{2}}{32}k_{5}A_{1}^{2}I_{11}^{2},$$

where *k*_1_≈1.29, *k*_4_≈2.29 and *k*_5_≈6.12—see (4.30) and (4.31) of Part 1. Substituting (A 4), one can verify that *L*_,*α*_, $L_{,\dot{\phi }}$, *M*_,*λα*_, $M_{,\lambda \dot{\phi }}$, $M_{,\alpha \phi \dot{\phi }}$ and $M_{,\phi {\dot{\phi }}^{2}}$ are slow functions of the aspect ratio, whereas *M*_,*ϕα*^2^_ behaves as *A*^−1^. For typical *A*=8 wing, all seven coefficients are of comparable magnitudes.

The aerodynamic model of Part 1 does not provide the contributions of the wing to the rate derivatives, $L_{,\dot{\tau }}$ and $M_{,\dot{\tau }}$. For high-aspect ratio (almost) un-swept wings, both contributions are invariably small [5, p. 137]. In dynamic stability analysis, $L_{,\dot{\tau }}$ is customarily neglected altogether. Towards the analysis carried out in this paper, the missing contribution to $M_{,\dot{\tau }}$can be easily compensated for by increasing respective contribution of the tail (see below)—it will not be needed, however.

Assuming that all wing sections have the same parasite drag coefficient, $D_{0}^{w}$, the wing's drag contribution to the pitching moment, *M*^*d*^, follows (3.3) by quadrature. For future use, it is put into the form:

A 13 $$M^{d}=-\frac{A}{4}\phi \int_{-1}^{1}\left|y\right|cD_{0}^{w}dy=M_{,D\phi }D_{0}^{w}\phi$$

where

A 14 $$M_{,D\phi }=-\frac{4}{3\pi }.$$

Taking the cue from fixed-wing airplanes [5], we postulate *M*^*bt*^ in the form

A 15 $$M^{\mathrm{bt}}=M_{,\dot{\tau }}\dot{\tau }+M_{,\alpha }^{\mathrm{bt}}(\tau -\dot{h})+M_{0}^{\mathrm{bt}}+M_{,\dot{\phi }}^{\mathrm{bt}}\dot{\phi },$$

where $M_{,\dot{\tau }}$ is the damping in pitch contributed mainly by the tail, $M_{,\alpha }^{\mathrm{bt}}$ is the stiffness in pitch contributed by the tail and the body alike, $M_{0}^{\mathrm{bt}}$ is the static moment contributed mainly by the tail, and $M_{,\dot{\phi }}^{\mathrm{bt}}$ reflects the reaction of the tail to the periodic downwash of the wing. None of these contributions will be explicitly required in the text; in fact, since the tail is normally closed in forward flight, one may expect *M*^*bt*^—and, consequently, all its partial derivatives—to be small for all but long-and-wide-bodied birds.

Combining (A 1), (A 5), (A 6), (A 14) and (A 15) together, yields

A 16 $$\begin{array}{rl} M & =M_{,\lambda \alpha }(\lambda +\dot{h}\phi )(\alpha_{g0}+\alpha )+M_{,\lambda \dot{\phi }}(\lambda +\dot{h}\phi )\dot{\phi }+M_{,\phi \alpha^{2}}\phi {\left(\alpha_{g0}+\alpha \right)}^{2}+L_{,\alpha }x_{\mathrm{cg}}(\alpha_{g0}+\alpha )+M_{,\dot{\tau }}\dot{\tau }\\ & \;+M_{,\alpha }^{\mathrm{bt}}\alpha +M_{,\alpha \phi \dot{\phi }}(\alpha_{g0}+\alpha )\phi \dot{\phi }+M_{0}^{\mathrm{bt}}+M_{,D\phi }D_{0}^{w}\phi +(M_{,\dot{\phi }}^{\mathrm{bt}}+L_{,\dot{\phi }}x_{\mathrm{cg}})\dot{\phi }+M_{,\phi {\dot{\phi }}^{2}}\phi {\dot{\phi }}^{2}, \end{array}$$

where, by interpretation, $\alpha =\tau -\dot{h}$ is the angle of attack of the bird's body.

Substituting (4.3) in (A 1) and (A 16), one will find that in the adjoint non-flapping flight

A 17 $$\bar{L}=L_{,\alpha }\alpha_{g,0}$$

and

A 18 $$\bar{M}=M_{,\lambda \alpha }\bar{\lambda }\alpha_{g0}+L_{,\alpha }x_{\mathrm{cg}}\alpha_{g0}+M_{0}^{\mathrm{bt}}.$$

Trim in this flight, namely,

A 19 $$\bar{M}=0,$$

is achieved with

A 20 $$\bar{\lambda }=-x_{\mathrm{cg}}\frac{L_{,\alpha }}{M_{,\lambda \alpha }}-\frac{M_{0}^{\mathrm{bt}}}{M_{,\lambda \alpha }\alpha_{g0}}=\frac{3\pi }{4}\left(x_{\mathrm{cg}}+\frac{M_{0}^{\mathrm{bt}}}{\bar{L}}\right);$$

the first part of (A 20) follows from (A 18) and (A 19); the last part follows from the first by (A 17) and (A 7). With (A 17)–(A 19), equations (A 1) and (A 16) can be rearranged as

A 21 $$L=\bar{L}+L_{,\alpha }\alpha +L_{,\dot{\phi }}\dot{\phi }$$

and

A 22 $$\begin{array}{rl} M & =(M_{,\lambda \alpha }(\alpha_{g0}+\alpha )+M_{,\lambda \dot{\phi }}\dot{\phi })(\lambda -\bar{\lambda }+\dot{h}\phi )+M_{,\phi \alpha^{2}}\phi \alpha (2\alpha_{g0}+\alpha )\\ & \;+{\bar{M}}_{,\alpha }\alpha +M_{,\dot{\tau }}\dot{\tau }+M_{,D\phi }{\bar{D}}^{w}\phi +{\bar{M}}_{,\dot{\phi }}\dot{\phi }+M_{,\alpha \phi \dot{\phi }}(\alpha_{g0}+\alpha )\phi \dot{\phi }+M_{,\phi {\dot{\phi }}^{2}}\phi {\dot{\phi }}^{2}, \end{array}$$

where

A 23 $$\begin{array}{rl} {\bar{M}}_{,\alpha } & =M_{,\alpha }^{\mathrm{bt}}+L_{,\alpha }x_{\mathrm{cg}}+M_{,\lambda \alpha }\bar{\lambda }=M_{,\alpha }^{\mathrm{bt}}+L_{,\alpha }\left(x_{\mathrm{cg}}-\frac{4}{3\pi }\bar{\lambda }\right), \end{array}$$

A 24 $$\begin{array}{rl} {\bar{M}}_{,\dot{\phi }} & =M_{,\dot{\phi }}^{\mathrm{bt}}+L_{,\dot{\phi }}x_{\mathrm{cg}}+M_{,\lambda \dot{\phi }}\bar{\lambda }=M_{,\dot{\phi }}^{\mathrm{bt}}+L_{,\dot{\phi }}\left(x_{\mathrm{cg}}-\frac{4}{3\pi }k_{1}\bar{\lambda }\right) \end{array}$$

A 25 $$\begin{array}{rl} \mathrm{and}\;\;{\bar{D}}^{w} & =D_{0}^{w}+\frac{M_{,\phi \alpha^{2}}}{M_{,D\phi }}\alpha_{g0}^{2}=D_{0}^{w}+\frac{{\bar{L}}^{2}}{\pi A}. \end{array}$$

In (A 23), the right-hand side follows by (A 7); in (A 24), the right-hand side follows by (A 8) and (A 3); in (A 25), it follows by (A 14), (A 11), (A 17) and (A 2). By interpretation, ${\bar{M}}_{,\alpha }$ is the pitch stiffness in the adjoint flight; consequently,

A 26 $${\bar{x}}_{n}=-\frac{M_{,\alpha }^{\mathrm{bt}}}{L_{,\alpha }}+\frac{4}{3\pi }\bar{\lambda }$$

is the respective neutral point (that is, the point where the pitch stiffness vanishes). ${\bar{D}}^{w}$ is the drag of the wing in the adjoint flight.

The following four combinations of partial derivatives:

A 27 $$\begin{array}{rl} M_{,\alpha \phi \dot{\phi }}^{\prime \prime } & =M_{,\alpha \phi \dot{\phi }}-2M_{,\phi \alpha^{2}}\frac{L_{,\dot{\phi }}}{L_{,\alpha }}, \end{array}$$

A 28 $$\begin{array}{rl} M_{,\phi {\dot{\phi }}^{2}}^{\prime \prime } & =M_{,\phi {\dot{\phi }}^{2}}-M_{,\alpha \phi \dot{\phi }}\frac{L_{,\dot{\phi }}}{L_{,\alpha }}+M_{,\phi \alpha^{2}}{\left(\frac{L_{,\dot{\phi }}}{L_{,\alpha }}\right)}^{2}, \end{array}$$

A 29 $$\begin{array}{rl} M_{,\lambda \dot{\phi }}^{\prime \prime } & =M_{,\lambda \dot{\phi }}-M_{,\lambda \alpha }\frac{L_{,\dot{\phi }}}{L_{,\alpha }} \end{array}$$

A 30 $$\begin{array}{rl} \mathrm{and}\;\;{\bar{M^{\prime \prime }}}_{,\dot{\phi }} & ={\bar{M}}_{,\dot{\phi }}-{\bar{M}}_{,\alpha }\frac{L_{,\dot{\phi }}}{L_{,\alpha }} \end{array}$$

naturally arise when grouping similar terms in (4.13); in view of (A 21), they can be interpreted as partial derivatives at constant *L*. Additional combination,

A 31 $${\bar{M}}_{,\dot{\phi }}^{\prime }={\bar{M}}_{,\dot{\phi }}+M_{,\dot{\tau }}\frac{L_{,\dot{\phi }}}{m}$$

arise when grouping $\dot{\phi }$ terms in (4.16); in view of (4.15), it can be interpreted as the respective derivative at constant *α*.

## Appendix B. Solutions of (5.7)

Consider the third-order differential equation with two time-dependent coefficients:

B 1 $$\frac{d^{3}x}{d{\tilde{t}}^{3}}+2\gamma_{s}\frac{d^{2}x}{d{\tilde{t}}^{2}}+(\kappa_{s}^{2}+2\psi_{1}^{2}\sin\tilde{t}-2\psi_{2}^{2}\sin2\tilde{t})\frac{dx}{d\tilde{t}}+(2\delta_{1}^{2}\sin\tilde{t}+2\delta_{2}^{2}\sin2\tilde{t})x=0,$$

in which *κ*_*s*_, *γ*_*s*_, *ψ*_1_, *ψ*_2_, *δ*_1_ and *δ*_2_ are constants. By analogy with Hill's equation [7, p. 405], we seek its solution in the form

B 2 $$x=\sum_{n=-\infty }^{\infty }\frac{b_{n}e^{i\left(\mu +i\gamma_{s}+n\right)\tilde{t}}}{i(\mu +i\gamma_{s}+n)}\;,$$

where *b*'s depend on the initial conditions and *μ* is Floquet's exponent [7, p. 414]. The factor $i\left(\mu +i\gamma_{s}+n\right)$, introduced in (B 2), is not a necessity, but it does make the following equations somewhat simpler.

*b*'s satisfy the recurrence relations

B 3 $$\begin{array}{rl} & \left(-i\psi_{2}^{2}+\frac{\delta_{2}^{2}}{\mu +i\gamma_{s}+n+2}\right)b_{n+2}+\left(i\psi_{1}^{2}+\frac{\delta_{1}^{2}}{\mu +i\gamma_{s}+n+1}\right)b_{n+1}+\left(-{\left(\mu +n\right)}^{2}+{\tilde{\kappa }}_{s}^{2}\right)b_{n}\\ & \;+\left(-i\psi_{1}^{2}-\frac{\delta_{1}^{2}}{\mu +i\gamma_{s}+n-1}\right)b_{n-1}+\left(i\psi_{2}^{2}-\frac{\delta_{2}^{2}}{\mu +i\gamma_{s}+n-2}\right)b_{n-2}=0, \end{array}$$

where ${\tilde{\kappa }}_{s}^{2}=\kappa_{s}^{2}-\gamma_{s}^{2}$; they follow by substitution of (B 2) in (B 1). These relations admit a non-trivial solution only if the determinant *H* of the infinite 5-diagonal matrix,

B 4 $$\begin{array}{rl} \left[H_{nm}\right] & =\frac{\mathrm{sgn}(m-n)\delta_{|m-n|,2}}{{\tilde{\kappa }}_{s}^{2}-n^{2}}\left(-i\psi_{2}^{2}+\frac{\delta_{2}^{2}}{\mu +i\gamma_{s}+m}\right)\\ & \;+\frac{\mathrm{sgn}(m-n)\delta_{\left|m-n\right|,1}}{{\tilde{\kappa }}_{s}^{2}-n^{2}}\left(i\psi_{1}^{2}+\frac{\delta_{1}^{2}}{\mu +i\gamma_{s}+m}\right)+\delta_{mn}\frac{{\tilde{\kappa }}_{s}^{2}-{\left(\mu +n\right)}^{2}}{{\tilde{\kappa }}_{s}^{2}-n^{2}}, \end{array}$$

formed on the coefficients of *b*'s in (5.9), vanishes. We will write this condition as

B 5 $$H\left(\mu ,{\tilde{\kappa }}_{s},\gamma_{s},\psi_{2},\psi_{1},\delta_{2},\delta_{1}\right)=0.$$

It determines *μ*.

We seek an analytical solution of (B 5). To this end, we form an auxiliary matrix,

B 6 $$\begin{array}{rl} \left[H_{nm}^{\prime }\right] & =\frac{\mathrm{sgn}\left(m-n\right)\delta_{\left|m-n\right|,2}}{{\tilde{\kappa }}_{s}^{2}-{\left(\mu +n\right)}^{2}}\left(-i\psi_{2}^{2}+\frac{\delta_{2}^{2}}{\mu +i\gamma_{s}+m}\right)\\ & \;+\frac{\mathrm{sgn}\left(m-n\right)\delta_{\left|m-n\right|,1}}{{\tilde{\kappa }}_{s}^{2}-{\left(\mu +n\right)}^{2}}\left(i\psi_{1}^{2}+\frac{\delta_{1}^{2}}{\mu +i\gamma_{s}+m}\right)+\delta_{mn}, \end{array}$$

related to [*H*_*nm*_] by

B 7 $$\left[H_{nm}\right]=\frac{{\tilde{\kappa }}_{s}^{2}-{\left(\mu +n\right)}^{2}}{{\tilde{\kappa }}_{s}^{2}-n^{2}}\left[H_{nm}^{\prime }\right].$$

Consequently,

B 8 $$H(\mu ,\ldots )=H^{\prime }(\mu ,\ldots )\prod_{n=-\infty }^{n=\infty }\frac{{\tilde{\kappa }}_{s}^{2}-{\left(\mu +n\right)}^{2}}{{\tilde{\kappa }}_{s}^{2}-n^{2}}=-H^{\prime }(\mu ,\ldots )\frac{\sin(\pi \mu -\pi {\tilde{\kappa }}_{s})\sin(\pi \mu +\pi {\tilde{\kappa }}_{s})}{\sin^{2}(\pi {\tilde{\kappa }}_{s})},$$

where *H*′ is the determinant of [*H*′_*nm*_] and the ellipses replace $\left\langle {\tilde{\kappa }}_{s},\gamma_{s},\psi_{2},\psi_{1},\delta_{2},\delta_{1}\right\rangle$. The infinite product appearing in (B 8) can be found in Whitaker & Watson [7, p. 416].

*H*′ is periodic with respect to *μ* with period 1—it can be verified by substituting *n*=*n*′+1 and *m*=*m*′+1 in (B 6). ${\left({\tilde{\kappa }}_{s}^{2}-\mu^{2}\right)}^{-1}$ and (*μ*+*iγ*_*s*_)^−1^ appear in the expansion of *H*′ only in the combinations ${\left({\tilde{\kappa }}_{s}^{2}-\mu^{2}\right)}^{-1}$ and ${\left({\tilde{\kappa }}_{s}^{2}-\mu^{2}\right)}^{-1}{\left(\mu +i\gamma_{s}\right)}^{-1}$—it follows from (B 6) because each term in the expansion of *H*′ contains one element from each row and one element from each column [10]. *H*′ tends to unity as μ→±i∞. With *δ*_1_=*δ*_2_=0 (and hence with no terms involving *γ*_*s*_), *H*′ turns into Hill's determinant. Compiling this information together, and taking the cue from the form of Hill's determinant [7, p. 416], we suggest that

B 9 $$\begin{array}{rl} H^{\prime }(\mu ,\ldots ) & =1+C_{1}(\cot(\pi \mu +\pi {\tilde{\kappa }}_{s})-\cot(\pi \mu -\pi {\tilde{\kappa }}_{s}))\\ & \;\;\;+\;C_{2}(\cot(\pi \mu +\pi {\tilde{\kappa }}_{s})-\cot(\pi \mu -\pi {\tilde{\kappa }}_{s}))\cot(\pi \mu +i\pi \gamma_{s}), \end{array}$$

where *C*_1_ and *C*_2_ are a pair of constants. Because (B 9) is periodic, if *C*_1_ and *C*_2_ can be adjusted to match the singularities of *H*′ at $\mu =\pm {\tilde{\kappa }}_{s}$ and *μ*=−*iγ*_*s*_, it will match all the singularities of *H*′, and, because (B 9) also matches *H*′ at infinity, it will match *H*′ at any *μ* by Liouville's theorem [7, p. 105].

With (B 9), equation (B 8) yields

B 10 $$H(\mu ,\ldots )=-\frac{\sin^{2}(\pi \mu )}{\sin^{2}(\pi {\tilde{\kappa }}_{s})}+1+2\cot(\pi {\tilde{\kappa }}_{s})C_{1}+2\cot(\pi {\tilde{\kappa }}_{s})C_{2}\cot(\pi \mu +i\pi \gamma_{s}),$$

or, what is equivalent,

B 11 $$H(\mu ,\ldots )=-\frac{\sin^{2}(\pi \mu )}{\sin^{2}(\pi {\tilde{\kappa }}_{s})}+B_{1}+B_{2}\frac{\cot(\pi \mu +i\pi \gamma_{s})}{\cot(i\pi \gamma_{s})},$$

where $B_{1}=1+2\cot(\pi {\tilde{\kappa }}_{s})C_{1}$ and $B_{2}=2\cot(\pi {\tilde{\kappa }}_{s})\cot(i\pi \gamma_{s})C_{2}$ replace *C*_1_ and *C*_2_ as the pair of independent parameters.

In general, *B*_1_ and *B*_2_ can be established by fitting (B 11) to *H* at two points, say, *μ*=0 and *μ*=*μ*_1_:

B 12 $$H(0,\ldots )=B_{1}+B_{2}$$

and

B 13 $$H(\mu_{1},\ldots )=-\frac{\sin^{2}(\pi \mu_{1})}{\sin^{2}(\pi {\tilde{\kappa }}_{s})}+B_{1}+B_{2}\frac{\cot(\pi \mu_{1}+i\pi \gamma_{s})}{\cot(i\pi \gamma_{s})}.$$

They yield

B 14 $$B_{2}=\frac{\cot(i\pi \gamma_{s})}{\cot(\pi \mu_{1}+i\pi \gamma_{s})-\cot(i\pi \gamma_{s})}\left(H(\mu_{1},\ldots )-H(0,\ldots )+\frac{\sin^{2}(\pi \mu_{1})}{\sin^{2}(\pi {\tilde{\kappa }}_{s})}\right)$$

and

B 15 $$B_{1}=H(0,\ldots )-B_{2}.$$

In practice, however, averaging *B*_2_ over four different points, uniformly distributed around zero, considerably reduces round-off errors; *B*_1_ still follows by (B 15). The result is shown in figure 7. An additional (indirect) indication that (B 11) is adequate is furnished below by solving (B 1) numerically and showing that its solution diverges where predicted by the present analysis and converges elsewhere.

Introducing (B 11) and

B 16 $$\mu =i\mu^{\prime }-i\gamma_{s}$$

in (B 5) yields

B 17 $$\frac{\sinh^{2}(\pi \mu^{\prime }-\pi \gamma_{s})}{\sin^{2}(\pi {\tilde{\kappa }}_{s})}+B_{1}+B_{2}\frac{\coth(\pi \mu^{\prime })}{\coth(\pi \gamma_{s})}=0.$$

Exploiting standard trigonometric identities, it can be rewritten as

B 18 $$w^{2}\frac{z^{2}+m^{2}-2mz}{m^{2}-1}+B_{1}+B_{2}\frac{m}{z}=0,$$

where

B 19 $$\begin{array}{rl} z & =\coth\left(\pi \gamma_{s}\right), \end{array}$$

B 20 $$\begin{array}{rl} m & =\coth\left(\pi \mu^{\prime }\right), \end{array}$$

B 21 $$\begin{array}{rl} \text{and}\;w^{2} & =\frac{\sinh^{2}(\pi \gamma_{s})}{\sin^{2}(\pi {\tilde{\kappa }}_{s})}. \end{array}$$

Essentially, (B 18) is a third-order equation for *m*:

B 22 $$B_{2}m^{3}+(B_{1}+w^{2})zm^{2}-(2w^{2}z^{2}+B_{2})m+(w^{2}z^{2}-B_{1})z=0.$$

It has three solutions; among them, those with Re(cosh−1⁡m)>0 are bounded.

Combinations of parameters for which all three solutions of (B 22) are bounded are shown in figure 1 in the text; part of figure 1*h* (with *γ*_*s*_=0.15) is reproduced in figure 8. Stability boundaries outlined in this figure were corroborated by direct numerical solution of (B 1). Initial conditions were set, rather arbitrarily, as

B 23 $$\frac{d^{2}x}{d{\tilde{t}}^{2}}=x=0,\;\frac{dx}{d\tilde{t}}=1.$$

The solution was examined at $\tilde{t}=400$ and classified as either diverging or converging. All solutions behaved as predicted.

## Appendix C. Numerical examples

Table 2 summarizes the details of all numerical examples shown in the paper.

**Table 2.** Numerical examples.

case	*A*	*ϕ*_0_	*Ω*	*α*_g0_	*ε*	$C_{D0}^{w}$	*C*_*L*,*α*_/*m*	*r*_*y*_	*x*_*cg*_	$\bar{\lambda }$
1	8	30	0.8	8	0.5	0.008	0.3	0.159	0	0
2	8	30	0.8	8	0.5	0.008	0.3	0.239	0	0
3	8	30	0.8	8	0.5	0.008	0.15	0.159	0	0
4	16	30	0.8	8	0.0	0.008	0.3	0.159	0	0
5	8	30	0.8	8	0.5	0.008	0.3	0.159	0.01	0
6	8	30	0.8	8	0.5	0.008	0.3	0.159	0.03	0.071
7	8	30	0.8	8	0.5	0.008	0.3	0.239	0.03	0.071
8	8	30	0.8	8	0.75	0.008	0.3	0.159	0.01	0
9	8	30	0.4	8	0.5	0.008	0.3	0.159	0.01	0
10	8	30	0.8	8	0.25	0.008	0.3	0.159	0.01	0
11	8	30	1.2	8	0.5	0.008	0.3	0.159	0.01	0

## Appendix D. Physical data

The data in this appendix were collected from the earlier studies ([4] and [11]; [12]; [13]; [14]); they are reproduced in the first six columns of table 3. In particular, *m** is the mass of a bird in kilograms, 2*s* and 2*S* are the span and the wing area in metres and square metres, respectively, *A* is the aspect ratio, *f** is the wing-beat frequency at cruise in cycles per second and *v* is the observed velocity at cruise in metres per second. *u** is the velocity at which the drag in the adjoint non-flapping flight is minimal; it was estimated with *u**=(*m***g*)^1/2^(*ρS*)^−1/2^(*πAD*_0_)^−1/4^, where *D*_0_ is the parasite drag coefficient [15]. The numbers shown in the table were based on *D*_0_=0.015 and standard density; one can verify that *u** provides a fair approximation for *v*. *u** replaced *v* when the latter was not included in the original data. In the following columns, *ω*=2*πf***s*/*v* is the reduced frequency, *m*=*m**/*ρSs* is the reduced mass; *L*_,*α*_ has been computed with (A 2). The next four columns reflect (5.21), (5.22), (5.14) and (5.15), respectively; *ψ*_2_ and *δ*_2_/*ψ*_2_ have been estimated with *ε*=0.5.

**Table 3.** Birds' data.

seq	scientific name	common name	*m**	2*s*	2*S*	*A*	*f**	*v*	*u**	*ω*	*m*	*L*_,*α*_/*m*	*L*_,*α*_/*mω*	$\Phi_{2}^{-1}$	$\Phi_{1}^{-2}$	*δ*_2_/*ψ*_2_	*δ*_1_/*ψ*_1_
1	*Pelecanoides georgicus*	South Georgia diving petrel	0.12	0.39	0.02	7.6	12.3		12.9	1.2	52.1	0.10	0.08	2.4	37.2	0.10	0.44
2	*Colaptes auratus*	northern flicker	0.13	0.51	0.05	5.4	9.2	12.7	9.3	1.2	17.6	0.26	0.23	2.1	41.6	0.16	0.65
3	*Pelecanoides urinatrix*	common diving petrel	0.13	0.41	0.02	7.5	12.3		12.7	1.2	48.2	0.10	0.08	2.5	39.9	0.10	0.45
4	*Pachyptila desolata*	dove prion	0.16	0.64	0.05	8.6	5.4	11.1	9.1	1.0	17.0	0.30	0.31	1.1	10.3	0.19	0.90
5	*Pica pica*	magpie	0.16	0.57	0.06	5.1	6.0	12.5	9.0	0.9	14.1	0.32	0.37	1.7	21.5	0.21	0.81
6	*Anas crecca*	teal	0.24	0.58	0.05	7.4	8.3	12.5	11.8	1.2	28.8	0.17	0.14	1.9	26.5	0.13	0.58
7	*Rynchops niger*	black skimmer	0.30	0.99	0.09	11.0	3.4	9.9	8.7	1.1	11.1	0.48	0.45	0.7	4.6	0.23	1.22
8	*Columba livia*	pigeon	0.32	0.62	0.06	6.2	6.0	12.5	12.2	0.9	26.5	0.18	0.19	2.0	18.5	0.15	0.62
9	*Larus atricilla*	laughing gull	0.33	1.03	0.11	10.0	2.7	9.5	8.5	0.9	9.7	0.54	0.58	0.7	3.6	0.25	1.32
10	*Egretta caerulea*	little blue heron	0.34	0.98	0.13	7.2	3.6	8.8	8.4	1.3	8.5	0.58	0.46	1.1	8.9	0.23	1.02
11	*Phaethon lepturus*	white-tailed tropicbird	0.37	0.92	0.09	10.1	4.2		10.1	1.2	15.4	0.34	0.28	1.0	7.5	0.18	0.92
12	*Rissa tridactyla*	kittiwake	0.39	0.97	0.10	9.2	3.2	13.1	9.6	0.7	13.0	0.40	0.54	0.8	4.9	0.25	1.23
13	*Stercorarius parasiticus*	Arctic skua	0.39	1.05	0.12	9.4	3.6	13.3	9.0	0.9	10.4	0.50	0.56	0.8	6.8	0.25	1.26
14	*Fratercula arctica*	puffin	0.40	0.55	0.04	8.2	9.2	17.6	16.7	0.9	64.0	0.08	0.09	2.2	27.1	0.10	0.47
15	*Daption capense*	Cape petrel	0.42	0.88	0.08	9.9	5.6	12.3	11.3	1.3	20.2	0.26	0.21	1.2	12.8	0.15	0.78
16	*Anas georgica*	South Georgia pintail	0.44	0.68	0.06	7.2	7.6		13.6	1.2	32.4	0.15	0.13	2.1	27.0	0.12	0.54
17	*Sterna maxima*	royal tern	0.47	1.15	0.11	12.2	3.1	10.0	9.6	1.1	12.4	0.44	0.39	0.7	4.1	0.21	1.18
18	*Chionis alba*	sheath bill	0.61	0.82	0.11	6.4	6.4		13.0	1.3	23.1	0.21	0.16	2.1	25.9	0.14	0.59
19	*Alca torda*	razorbill	0.62	0.66	0.05	9.5	9.1	16.0	18.0	1.2	66.6	0.08	0.07	2.2	26.8	0.09	0.44
20	*Fulmarus glacialis*	fulmar	0.82	1.13	0.12	10.3	4.6	13.0	12.3	1.3	19.0	0.28	0.22	1.1	10.6	0.16	0.83
21	*Casmerodius albus*	great egret	0.87	1.34	0.22	8.1	2.8	10.6	10.1	1.1	9.6	0.53	0.47	1.0	6.2	0.23	1.09
22	*Larus dominicanus*	kelp gull	0.89	1.41	0.23	8.7	3.5		9.9	1.6	9.0	0.57	0.36	1.0	9.1	0.20	0.99
23	*Eudocimus albus*	white ibis	0.90	0.95	0.16	5.7	4.7	12.9	13.2	1.1	19.3	0.24	0.22	2.0	18.9	0.16	0.65
24	*Uria aalge*	common guillemot	0.95	0.71	0.05	9.2	8.7	19.1	20.7	1.0	81.3	0.06	0.06	2.3	27.2	0.08	0.42
25	*Larus argentatus*	herring gull	0.95	1.36	0.20	9.1	3.1	9.9	10.7	1.3	11.2	0.46	0.35	1.0	6.5	0.20	0.98
26	*Procellaria aequinoctialis*	white-chinned petrel	1.23	1.41	0.17	11.9	3.9		12.5	1.4	17.1	0.32	0.23	0.9	8.2	0.16	0.89
27	*Anhinga anhinga*	anhinga	1.24	1.17	0.17	7.9	5.1		13.7	1.4	20.0	0.25	0.18	1.6	18.3	0.15	0.68
28	*Ajaia ajaja*	roseate spoonbill	1.30	1.25	0.23	6.9	3.9	11.9	12.7	1.3	15.0	0.32	0.25	1.5	13.6	0.17	0.75
29	*Catharacta skua*	great skua	1.35	1.37	0.21	8.8	3.9	14.9	12.5	1.1	15.0	0.34	0.30	1.1	11.4	0.18	0.90
30	*Phalacrocorax auritus*	double-crested cormorant	1.41	1.16	0.18	7.5	5.0	14.5	14.6	1.3	22.2	0.22	0.18	1.7	19.0	0.14	0.65
31	*Fregata magnificens*	magnificent frigatebird	1.47	2.29	0.41	12.9	2.2	9.3	8.6	1.7	5.1	1.06	0.61	0.5	4.0	0.26	1.51
32	*Pandion haliaetus*	osprey	1.49	1.59	0.30	8.4	3.3	10.6	11.2	1.6	10.2	0.50	0.32	1.1	9.8	0.19	0.91
33	*Larus marinus*	great black-backed gull	1.55	1.65	0.29	9.6	2.9	13.0	11.4	1.2	10.8	0.48	0.42	0.9	6.8	0.22	1.10
34	*Cathartes aura*	turkey vulture	1.55	1.75	0.44	6.9	3.0	10.6	9.9	1.6	6.5	0.75	0.48	1.1	11.2	0.24	1.04
35	*Phalacrocorax aristotelis*	shag	1.81	1.04	0.16	6.8	5.4	15.4	18.0	1.1	36.0	0.14	0.12	2.3	21.6	0.12	0.51
36	*Ardea Herodias*	great blue heron	1.92	1.76	0.42	7.4	2.6	9.4	11.2	1.5	8.5	0.58	0.39	1.2	7.6	0.21	0.95
37	*Coragyps atratus*	black vulture	2.08	1.38	0.33	5.8	4.5	10.8	13.9	1.8	15.1	0.31	0.17	2.2	25.1	0.14	0.58
38	*Phalacrocorax atriceps*	blue-eyed shag	2.23	1.13	0.18	7.0	5.9		18.4	1.1	35.2	0.14	0.12	2.2	27.4	0.12	0.53
39	*Ardea occidentalis*	great white heron	2.50	1.91	0.49	7.4	2.7	11.0	11.7	1.5	8.7	0.57	0.39	1.2	9.0	0.21	0.96
40	*Sula bassanus*	northern gannet	3.01	1.85	0.26	13.1	3.5	14.9	15.3	1.4	20.3	0.27	0.20	0.9	7.8	0.15	0.86
41	*Diomede melanophris*	black-browed albatross	3.08	2.19	0.35	13.5	3.0	13.3	13.2	1.5	13.0	0.42	0.27	0.7	6.3	0.17	1.03
42	*Macronectes giganteus*	giant petrel	3.24	1.98	0.33	12.0	3.1	15.2	14.5	1.3	16.4	0.33	0.26	0.9	7.3	0.17	0.95
43	*Pelecanus occidentalis*	brown pelican	3.39	2.26	0.45	11.4	3.0	10.1	12.8	2.1	10.9	0.49	0.23	1.0	8.2	0.16	0.88
44	*Haliaeetus leucocephalus*	bald eagle	4.68	2.24	0.76	6.6	2.7	11.2	13.3	1.7	9.0	0.54	0.31	1.4	12.5	0.19	0.82
45	*Cygnus Cygnus*	whooper swan	8.50	2.26	0.59	8.7	3.6		19.0	1.3	20.9	0.24	0.18	1.4	15.6	0.14	0.70
46	*Diomedea exulans*	wandering albatross	8.55	3.01	0.58	15.5	2.5	15.0	16.6	1.6	15.9	0.35	0.22	0.7	5.2	0.16	0.99

The choice of 0.015 for *D*_0_ was somewhat arbitrary, as the actual value of the drag coefficient depends on the shape of the bird's body and its size relative to the wing; moreover, *D*_0_ changes with the Reynolds number. Preliminary design tools found in reference [16] suggest that a tailless airplane, featuring an elliptical wing with aspect ratio of eight, and a double-ogive body, two root-chords long and a half root-chord in diameter, should have *D*_0_ between 0.015 and 0.018 at chord-based Reynolds numbers between 200 000 and 80 000. This estimate is based on the assumption that the boundary layer on its surface is turbulent. It drops by half if the boundary layer can be assumed laminar.
